# Diagnosis and management of non-alcoholic fatty liver disease and related metabolic disorders: Consensus statement from the Study Group of Liver and Metabolism, Chinese Society of Endocrinology

**DOI:** 10.1111/1753-0407.12056

**Published:** 2013-06-04

**Authors:** Xin Gao, Jian-Gao Fan

**Affiliations:** 1 Department of Endocrinology and Metabolism Zhongshan Hospital, Fudan UniversityShanghai, China; 2 Department of Gastroenterology, Xinhua HospitalShanghai, China

**Keywords:** NAFLD, metabolic disorders, diagnosis, management

## Abstract

Non-alcoholic fatty liver disease (NAFLD) is the most common liver disease in Western countries, affecting 20%–33% of the general population. Large population-based surveys in China indicate a prevalence of approximately 15%–30%. Worldwide, including in China, the prevalence of NAFLD has increased rapidly in parallel with regional trends of obesity, type2 diabetes and metabolic syndrome. In addition, NAFLD has contributed significantly to increased overall, as well as cardiovascular and liver-related, mortality in the general population. In view of rapid advances in research into NAFLD in recent years, this consensus statement provides a brief update on the progress in the field and suggests preferred approaches for the comprehensive management of NAFLD and its related metabolic diseases.

## Introduction

Non-alcoholic fatty liver disease (NAFLD) is defined as liver fat accumulation exceeding 5% by weight that is not the result of excessive alcohol consumption, drugs, toxins, infectious diseases or any other identifiable causes. It is a spectrum of disorders ranging from simple fatty liver (NAFL) to non-alcoholic steatohepatitis (NASH) with or without fibrosis/cirrhosis.^1^ Even a small proportion of NAFLD patients can advance to hepatocellular carcinoma (HCC). Histologically, NAFL is defined as the presence of hepatic steatosis with no evidence of hepatocellular injury (e.g. the form of ballooning hepatocytes), whereas NASH is defined as the presence of hepatic steatosis and inflammation with hepatocyte injury, with or without fibrosis.

In Western countries, NAFLD is the most common liver disorder, affecting 20%–33% of the general population.^2^,^3^ In patients with obesity and diabetes, the prevalence of NAFLD is 57.5%–74.0% and 21–78%, respectively.^4^ There is strong evidence that the prevalence of NAFLD has been increasing rapidly in the Asia Pacific region in recent years, approaching levels seen in Western countries.^5^ In China, the prevalence of NAFLD is also of concern. The prevalence of NAFLD has been found to be approximately 15% in Shanghai^6^ and nearly 30% in Hong Kong, as determined by accurate proton magnetic resonance spectroscopy.^7^

The risk factors for NAFLD include changes in lifestyle, notably the adoption of a high-fat diet and physical inactivity, and the presence of metabolic syndrome or its components, including central abdominal obesity, hypertension, dyslipidemia and type 2 diabetes (T2D).^8^ It is likely that NAFLD is the hepatic manifestation of metabolic syndrome where insulin resistance is the main risk factor.^2^ Many recent studies have reported that the increase in the prevalence of NAFLD is associated with increases in the prevalence of prediabetes, diabetes, and cardiovascular disease.^9^,^10^ There is also evidence that the prevalence of NAFLD prevalence is affected by age, gender, ethnicity, and other pathological status, such as hypothyroidism, hypopituitarism, hypogonadism, sleep apnea, and polycystic ovary syndrome.^11^,^12^

There is a strong association between NAFLD and multiple metabolic diseases, which is traditionally thought to be due, in part, to the same causal factors comprising both genetic and environmental factors. However, there is accumulating evidence that NAFLD and its related metabolic diseases pathogenically interact with each other through specific mechanisms. Recent epidemiologic studies have shown that NAFLD and its severity independently predict the occurrence of T2D and cardiovascular diseases.^13^–^15^ Conversely, it is well recognized that the presence of metabolic syndrome or diabetes is a strong predictor for the presence of steatohepatitis and fibrosis in NAFLD patients.^16^–^18^ It is estimated that in T2D patients, the prevalence of NASH reaches 63%–78%, whereas that of fibrosis is in the range 22%–60%;^18^ NASH and/or fibrosis is found in approximately 40% of patients with metabolic syndrome.^17^ Such a tight connection between NAFLD and metabolic diseases suggests that NAFLD is not a disease that occurs in the liver only. Thus, NAFLD and associated metabolic diseases should be managed in a comprehensive manner to improve both liver and global outcomes. To this end, our group, which comprises experts in the fields of endocrinology, diabetes, and gastroenterology, worked together to reach a consensus regarding the management of NAFLD and related metabolic disorders from a clinical aspect. Wherever possible the recommendations are evidence based and, when such evidence is not available or is inconsistent, the recommendations are made on the basis of a consensus opinion from the association members. This consensus statement has adopted the classification for evidence quality and recommendation strength used by the Grading of Recommendation Assessment, Development, and Evaluation (GRADE) workgroup (http://www.uptodate.com/home/grading-guide, accessed 10 January 2013) with minor revisions (Table 1). The strength of recommendations is classified as strong (1) or weak (2), whereas the quality of the evidence supporting strong or weak recommendations is designated as being high (A), moderate (B) or low (C). We expect that this consensus statement will help clinicians make comprehensive decisions from both the diagnostic and therapeutic viewpoints based on a full understanding of the best currently available clinical evidence, medical resources, and full consideration of the specific condition and wishes of individual patients.

**Table 1:** Grading of recommendations, assessment, development and evaluation (GRADE)^61^

	Criteria
Strength of recommendation	
Strong (1)	Factors influencing the strength of the recommendation include the quality of the evidence, presumed patient-important outcomes, and cost
Weak (2)	Variability in preferences and values, or more uncertainty Recommendation is made with less certainty, higher cost, or resource consumption
Quality of evidence	
High (A)	Further research is unlikely to change confidence in the estimate of the clinical effect
Moderate (B)	Further research may change confidence in the estimate of the clinical effect
Low (C)	Further research is very likely to impact on the confidence on the estimate of the clinical effect

## Definition

Non-alcoholic fatty liver disease is characterized by: (i) the presence of hepatic steatosis, as determined by imaging or histological diagnosis; (ii) no history of excessive alcohol drinking or the consumption of <140 g/week ethanol intake for men (<70 g/week for women) in the past 12 months; and (iii) no competing etiologies for hepatic steatosis and no coexisting causes for chronic liver disease.^19^

Because a pathological liver diagnosis is often not possible for epidemiological studies or in the clinical setting, a practical definition of NAFLD is required. Thus, we propose a working definition of NAFLD of: (i) results of liver imaging meet the diagnostic criteria of diffuse fatty liver that cannot be explained by any other causes; or (ii) an unexplained consistent increase in serum alanine aminotransferase (ALT), aspartate aminotransferase (AST) and/or glutamyl transpeptidase (GGT) levels for at least 6 months in patients with any component of the metabolic syndrome. A definitive diagnosis of NAFLD can be made if elevated liver enzyme levels and changes on fatty liver imaging improve or even return to normal after successful reduction in body weight and improvement in insulin resistance.^20^

## Screening

Clinically, most NAFLD patients are asymptomatic, although some may present with fatigue, dyspepsia, dull pain in the liver, and hepatosplenomegaly. The patients are often overweight or obese and present with manifestations of diabetes and other components of metabolic syndrome. It can be argued that a systematic screening for NAFLD is recommended, at least among higher-risk individuals in diabetes or obesity clinics. Because elevated liver enzymes are only detected in approximately 20% of NAFLD patients, these studies may not be sensitive enough to serve as a screening test. Liver ultrasound examination is potentially more sensitive and relatively cheap and is commonly used as a screening test.

### Recommendation

- Ultrasound examination-based screening for NAFLD in high-risk adults, especially those who attend diabetes or obesity clinics, is advised (Strength 1; Evidence B)

## Diagnosis

For patients who are found to have hepatic steatosis during a screening test or as an incidental discovery, it is necessary to perform investigations to rule out alternative causes, such as significant alcohol consumption or medications, before a diagnosis of NAFLD can be established.

### Exclusion of secondary causes of steatosis

#### Exclusion of alcohol consumption

Alcohol consumption in patients with suspected NAFLD is defined as the consumption of <20 g ethanol/day in men (<140 g/week) and 10 g ethanol/day in women (<70 g/week).^20^ Daily alcohol consumption is calculated as: (volume of alcohol consumed per day (mL) × alcohol concentration (%) × 0.8).

### Exclusion of other liver diseases

#### Patients with secondary causes of steatosis and elevated liver enzymes

All chronic liver diseases (including viral hepatitis, autoimmune hepatitis, celiac disease, hepatolenticular degeneration, α1-antitrypsin deficiency), hepatic malignancies, hepatobiliary infections diseases, and biliary tract diseases should be excluded before ascribing abnormal liver tests to NAFLD. In hepatitis B s antigen (HBsAg)-positive patients with serum hepatitis B virus (HBV) DNA titers below 10^4^ copies/mL, increases in liver enzymes are more likely to be due to fatty liver disease if metabolic risk factors are present.^20^ A moderate increase in serum ferritin levels is common in patients with NAFLD and it does not necessarily indicate increased iron stores. Patients with obvious increases in serum ferritin and transferrin saturation who are suspected of having NAFLD should undergo additional tests for genetic hemochromatosis.^21^ The C282Y mutation of the hemochromatosis (HFE) gene is rare and its clinical significance is unclear in Chinese populations.^22^ A recent large study from the NASH Clinical Research Network reported that 21% of patients with well-phenotyped NAFLD are positive for serum autoantibodies.^23^ However, the link between serum autoantibodies and NAFLD seems weak in Chinese populations.^24^ Therefore, a complete differentiation for autoimmune liver disease should be considered in patients with high serum titers of autoantibodies.

### Exclusion of medications that cause steatosis

The effects of drugs that cause hepatic steatosis, such as estrogen, tamoxifen, amiodarone, sodium valproate etc., should be carefully excluded before diagnosing NAFLD.^25^

### Exclusion of systemic diseases

Fatty liver caused by whole-body systemic diseases, including total parenteral nutrition (TPN), inflammatory bowel disease (IBD), hypopituitarism, hypothyroidism, and lipoatrophy, is often accompanied by liver steatosis. Therefore, the preferred nomenclature should include the known causative factor and the resultant pathology; for example, TPN-induced NAFLD (or NASH) rather than “secondary fatty liver diseases”.^20^

### Recommendations

- In patients with hepatic steatosis detected on imaging examinations, it is essential to perform investigations to rule out alternative causes and exclude coexisting common chronic liver disease (Strength 1; Evidence A).
- In HBsAg-positive patients with serum HBV DNA titers below 10^4^ copies/mL, elevated liver enzymes are more likely to be due to fatty liver disease if metabolic risk factors are present (Strength 2; Evidence B)
- A persistently high level of serum ferritin and increased iron saturation may warrant a liver biopsy, and a C282Y mutation in the *HFE* gene may not help diagnosis in the Chinese population (Strength 1; Evidence B)
- High serum titers of autoantibodies in association with other features suggestive of autoimmune liver disease (very high aminotransferases, high globulin) should prompt a more complete work-up for autoimmune liver disease (Strength 1; Evidence B)

## Clinical features

Symptoms such as fatigue, dyspepsia, dull pain in the liver, and hepatosplenomegaly are non-specific and rarely seen in NAFLD patients. The presence of NAFLD should be suspected in patients with metabolic risk factors (e.g. central obesity, T2D, dyslipidemia, metabolic syndrome) and imaging findings of hepatic steatosis.

### Recommendation

- NAFLD can be diagnosed if: (i) hepatic imaging results are compatible with fatty liver; (ii) secondary causes are ruled out; and (iii) metabolic risk factors are present (Strength 1; Evidence A)

## Liver enzyme examinations

Slightly elevated ALT and AST levels (i.e. 1.5–2-fold the upper limit of normal) with no obvious causes may be an indicator of NAFLD. However, we cannot rely on liver enzyme examinations alone because liver enzyme levels are often normal in most NAFLD patients. In addition, liver enzyme levels fluctuate with the development of liver disease and may even be normal at advanced stages of cirrhosis. Thus, there are obvious limitations to the use of liver enzymes as markers for the diagnosis and monitoring of the activity of NAFLD.^26^

### Recommendation

- Serum liver enzyme examinations are not sensitive enough to diagnose NAFLD and are susceptible to interference from other clinical conditions (Strength 1; Evidence A)

## Imaging diagnosis

Imaging results showing fat accumulation in the liver are necessary for a diagnosis of NAFLD. Currently, methods based on ultrasonography, computed tomography (CT), and magnetic resonance imaging (MRI) are most often used, with ultrasound the most common technique used in clinical practice to diagnose fatty liver due because of its simplicity, low cost, non-invasiveness, and good applicability. In addition, ^1^H-magnetic resonance spectroscopy (^1^H-MRS) can accurately measure hepatic fat content non-invasively. However, none of these imaging modalities can reliably assess steatohepatitis and fibrosis in patients with NAFLD.

### Abdominal ultrasonography

Based on the guidelines for the assessment and management of NAFLD in the Asia Pacific region,^27^ fatty liver can be diagnosed by the presence of at least two of the following three abnormal findings on abdominal ultrasonography: (i) increased echogenicity of the liver near-field region with deep attenuation of the ultrasound signal; (ii) hyperechogenity of liver tissue (“bright liver”), as often compared to hypoechogenity of the kidney cortex; and (iii) vascular blurring.

The following should be taken into consideration when using ultrasonography to diagnose fatty liver.

- For severely obese individuals, the sensitivity and specificity of ultrasonography in detecting NAFLD falls to 49% and 75%, respectively, possibly due to image blurring caused by thickening of the abdominal subcutaneous and visceral fat.
- For patients with a hepatic fat content <20%, the sensitivity of ultrasonography for the diagnosis of NAFLD is only 55%.
- Increased echogenicity of liver can be also present on ultrasound images of hepatic fibrosis, leading to a misdiagnosis of fatty liver.

In addition, there is significant variability in ultrasound results between different operators and between different ultrasound machines.^28^

### Computed tomography

Fatty tissues are less dense and appear darker than fat-free tissues on unenhanced CT. The assessment of hepatic steatosis by unenhanced CT can be made by determining either absolute hepatic attenuation (≤40 Hounsfield units [HU])^29^ or the ratio of the liver to spleen CT attenuation (L/S ratio; ≤1). The L/S ratio is often well correlated with the degree of liver steatosis and can be used to briefly evaluate the severity of fatty liver (i.e. for mild, moderate and severe steatosis, the L/S ratio is 0.7–1, 0.5–0.7, and ≤0.5, respectively^30^).

The use of CT is associated with radiation exposure, which limits its application in longitudinal studies and in children. Moreover, a CT examination is a qualitative diagnostic method for fatty liver with a very poor sensitivity for the diagnosis of mild hepatic steatosis (liver fat content <30%). Therefore, CT has no advantages over ultrasonography in terms of detecting mild hepatic steatosis, monitoring the progression of the disease, and evaluating the efficacy of treatment.^31^ As such, CT is not recommended as a routine modality for the diagnosis of NAFLD.

### ^1^H-Magnetic resonance spectroscopy

The theoretical basis for ^1^H-MRS determination of liver fat content is the difference between the fat and water ^1^H spectrum in a single voxel of liver tissue.^32^ Currently, ^1^H-MRS is recognized as the only accurate non-invasive method for the quantification of hepatic fat content. It provides a sensitive method for the early detection of mild NAFLD and evaluation of the efficacy of treatment. It is also a valuable technique for large-scale quantitative studies on NAFLD and other relevant metabolic disorders.^32^ However, ^1^H-MRS examinations are expensive and, until now, it has remained a research tool, although this may change with the propagation of this technique. There has also been a significant interest in developing simple quantitative imaging methods for the determination of hepatic fat content,^33^ but the detailed discussion of these methods is beyond the scope of the present consensus statement.

### Recommendations

- Ultrasonography is recommended as the currently most appropriate imaging modality for NAFLD screening. At least two of three abnormal manifestations on abdominal ultrasonography are required for a diagnosis of steatosis. The limitations of ultrasound-based examination (including inter-and intra-observer variability, poor sensitivity in detecting mild hepatic steatosis, and lack of specificity) should be taken into consideration in clinical practice (Strength 1; Evidence A)
- CT has no major advantages over ultrasonography in terms of diagnostic accuracy and is not recommended because of its expense and radiation exposure (Strength 1; Evidence A)
- ^1^H-MRS can quantify hepatic fat content accurately, which can be very useful in assessing the efficacy of therapeutic interventions, but it is expensive and not widely available. If conditions permit, ^1^H-MRS is recommended for patients suspected of having NAFLD diagnosis is ambiguous or for those patients who need an accurate and sensitive assessment of the progression of NAFLD (Strength 1; Evidence B)

## Non-invasive assessment of steatohepatitis and advanced fibrosis in NAFLD

Generally, NAFL is benign, whereas NASH can progress to cirrhosis, liver failure, and liver cancer^1^ and has a high liver-related mortality rate. It is necessary to identify the presence of steatohepatitis and fibrosis in patients with NAFLD. Liver biopsy is recognized as the most reliable approach for a pathological diagnosis of steatohepatitis, but its use is limited by cost, sampling errors, and procedure-associated morbidity and mortality.

There has been intense interest in developing non-invasive methods to identify advanced fibrosis in patients with NAFLD, including establishment of the NAFLD fibrosis score, the enhanced liver fibrosis (ELF) panel, and the use of transient elastography.^34^ The NAFLD fibrosis score is based on six readily available variables (i.e. age, body mass index (BMI), hyperglycemia, platelet count, albumin, and AST : ALT ratio) and is recognized as a clinically useful tool to identify NAFLD patients with a higher likelihood of having bridging fibrosis and/or cirrhosis, as described in recent US guidelines.^35^ Studies using the NAFLD fibrosis score in Chinese populations demonstrated that it had a 88%–91% negative predictive value in excluding advanced fibrosis and could reduce the burden of liver biopsy in most NAFLD patients.^36^,^37^ The ELF panel consists of assays for plasma levels of hyaluronic acid, tissue-specific inhibitor of metalloproteinase (TIMP)-1, and procollagen III N-terminal propeptide (PIIINP), but it is not currently available commercially in China.^35^ Transient elastography is a non-invasive measure of liver stiffness that has high sensitivity and specificity for identifying fibrosis in NAFLD patients.^34^ However, transient elastography has high failure rate in obese individuals and is not commercially available in most medical institutions.

### Recommendation

- The NAFLD fibrosis score can reliably exclude advanced fibrosis in Chinese NAFLD patients and is useful for avoiding unnecessary liver biopsy for most NAFLD patients (Strength 1; Evidence B)

## Liver biopsy

Liver biopsy remains the gold standard for characterizing liver histology in patients with NAFLD. However, a diagnosis of NAFLD in the clinic may not always rely on biopsy results because of the high cost and potential injuries associated with the procedure. Liver biopsy for the diagnosis of NAFLD can be considered under the following conditions: (i) the diagnosis of NAFLD is ambiguous; (ii) the patients are at high risk of steatohepatitis and advanced fibrosis (accompanied by metabolic syndrome or an NAFLD fibrosis score ≥–1.455); and (iii) for patients who are involved in special clinical trials.^20^

According to the NAFLD guidelines suggested by the Society of Liver Disease, Chinese Medical Association,^38^ NAFLD can be classified into simple steatosis, NASH and NASH-related cirrhosis. The NAFLD activity score (NAS) and fibrosis score established by the US National Institutes of Health Non-alcoholic Steatohepatitis Clinical Research Network (NASH CRN) is recommended for initial diagnosis and for use in therapeutic trials.^39^

### Simple steatosis

Depending on the extent of hepatocellular steatosis, simple fatty liver can be categorized into four grades based on the steatosis score (0–3): 0, hepatocellular steatosis <5%; 1, hepatocellular steatosis between 5% and 33%; 2, hepatocellular steatosis between 33% and 66%; and 3, hepatocellular steatosis >66%.^39^

### Non-alcoholic steatohepatitis

The NAS provides a single score to categorize liver biopsies as NASH, borderline NASH, and no NASH. The total NAS score represents the sum of scores ranging from 0 to 8 for steatosis, lobular inflammation, and ballooning. The categorization of the extent of hepatocellular steatosis in NASH is similar to that of simple fatty liver. The degree of lobular inflammation in NASH is categorized into four grades (lobular inflammation score 0–3) as follows: 0, no inflammatory foci; 1, fewer than two foci per ×200 field; 2, two to four foci per ×200 field; and 3, more than four foci per ×200 field. Ballooning of NASH can be divided into three categories (hepatocyte ballooning score 0–2) as follows: 0, no balloon cells; 1, a few balloon cells; 2, many balloon cells or prominent ballooning. An NAS <3 is largely considered to indicate no NASH, whereas patients with an NAS >4 are diagnosed as having NASH. Those patients with scores between 3 and 4 are diagnosed as probably having NASH. The stage of fibrosis is evaluated separately and divided into five categories (fibrosis stage 0–4) as follows: Stage 0, no fibrosis; Stage 1, perisinusoidal or periportal fibrosis (1A, mild zone 3, perisinusoidal fibrosis; 1B, moderate, zone 3, perisinusoidal fibrosis; 1C, portal/periportal fibrosis); Stage 2, perisinusoidal and portal/periportal fibrosis; Stage 3, bridging fibrosis; Stage 4, cirrhosis.^39^

### Non-alcoholic steatohepatitis-related cirrhosis

In NASH-related cirrhosis, the entire normal structure of the liver is destroyed and replaced by pseudolobules and widespread fibrosis, which appears macroscopically as small nodular cirrhosis. NASH-related liver cirrhosis can be further classified as active or static cirrhosis depending on whether there is interface hepatitis in the fibrotic septum.

### Recommendations

- The existence of metabolic syndrome and having a high NAFLD fibrosis score may be indicators of patients who are at risk of steatohepatitis and advanced fibrosis; in these patients, liver biopsy should be considered (Strength 1; Evidence B)
- Liver biopsy should be also considered in patients with suspected NAFLD or those with evident etiologies for hepatic steatosis and in whom coexisting chronic liver diseases cannot be excluded (Strength 1; Evidence B)
- Routine assessment of the NAS and liver fibrosis stage is recommended for the pathological diagnosis of NAFLD (Strength 1; Evidence B)

## Evaluation of NAFLD-associated metabolic disorders

It is generally agreed that patients with simple steatosis have very slow, if any, histological progression, whereas patients with NASH can exhibit histological progression to cirrhotic-stage disease. Currently, the estimated prevalence of NASH is low, ranging from 3% to 5%.^11^ Thus, the liver prognosis is not the main concern for most NAFLD patients. However, 48.8% of NAFLD patients also have metabolic syndrome and up to 50% fulfill the diagnostic criteria of prediabetes or diabetes in China.^40^ Studies on long-term outcomes have reported that patients with NAFLD have increased overall mortality compared with matched control populations, and that the most common cause of death in patients with NAFLD, NAFL, and NASH is cardiovascular disease.^41^,^42^ Therefore, we strongly suggest that a comprehensive assessment of the metabolic state, as well as risks for diabetes and cardiovascular diseases, is undertaken in patients with NAFLD.

The metabolic evaluations should include the following.

- Anthropometry (i.e. routine measurements of height, weight, BMI, and waist circumference).
- Glucose metabolism. Assays of fasting and 2-h postprandial blood glucose (fasting blood glucose [FBG] and post-load blood glucose [PBG]) levels are recommended as screening examinations. When FBG is ≥5.6 mmol/L or PBG is ≥7.8 mmol/L, an oral glucose tolerance test (OGTT; 75 g glucose) and HbA1c assays are recommended. A hyperglycemic clamp is recommended only for clinical research. If a diagnosis of diabetes is confirmed, systemic evaluation of diabetic complications is recommended, including risk factors of cardiovascular disease (blood pressure, lipid profile, smoking), urinary albumin excretion, retinal photography, and signs and symptoms of neuropathy, according to the 2013 American Diabetes Association (ADA) guidelines.^43^ Imaging examinations for coronary heart disease (CHD) are not recommended as a screening test for asymptomatic NAFLD patients with diabetes.
- Lipid metabolism. NAFLD is associated with a broad spectrum of lipid abnormalities. Serum triglyceride (TG) and high-density lipoprotein–cholesterol (HDL-C) levels should be measured at the time of diagnosis of NAFLD. Dyslipidemia is defined as TG levels >150 mg/dL (1.7 mmol/L) or HDL-C <40 mg/dL (0.9 mmol/L) in men and <50 mg/dL (1.1 mmol/L) in women according to the 2005 criteria of the International Diabetes Federation (IDF).^44^
- Blood pressure. Blood pressure measurement is recommended for NAFLD patients, with a blood pressure target of 140/90 mmHg (or 130/80 mmHg in patients with diabetes and/or renal dysfunction).
- Other concomitant endocrine diseases. Endocrine disease-related secondary hepatic steatosis should be carefully excluded in NAFLD patients with any signs or symptoms of the following endocrine disorders: polycystic ovary syndrome, Cushing syndrome, adrenal insufficiency, hypothyroidism, or hypopituitarism.
- Cardiovascular risks for NAFLD patients. Cardiovascular disease is the leading cause of death in NAFLD patients, and NAFLD is associated with increased vascular risk independent of conventional cardiometabolic risk factors.^4^,^9^ It is expected that early evaluation of cardiovascular risks and subsequent early intervention will improve the survival rate for NAFLD patients. The carotid intima–media thickness (CIMT) is increasingly used as a surrogate marker for atherosclerosis^45^ and helps clinicians more effectively identify vulnerable patients who would benefit from aggressive preventative intervention.^46^ A recent review article reported that NAFLD patients have increased CIMT and suggested that routine CIMT measurements should be implemented in NAFLD patients.^47^

### Recommendations

- Once a diagnosis of NAFLD is established, the patient's metabolic state and cardiovascular risks should be evaluated (Strength 1; Evidence A)
- NAFLD patients with newly diagnosed diabetes require systemic evaluation for the existence of diabetic complications (Strength 1; Evidence B)
- Routine measurement of CIMT is recommended for NAFLD patients as a surrogate marker of atherosclerosis (Strength 2; Evidence B)

## Treatment of NAFLD

The management of patients with NAFLD consists of treating the liver disease as well as the associated metabolic disorders, including obesity, insulin resistance, hyperlipidemia, and T2D.

### Lifestyle interventions

For overweight or obese (abdominal obesity) NAFLD patients, lifestyle interventions aiming to reduce body weight are considered as the fundamental treatment. NAFLD patients should be educated to control their dietary intake, increase their physical activity, and change their unhealthy lifestyle.

#### Recommendations

- Exercise. Moderate aerobic exercise at least four times a week, with a minimum total exercise time of 150 min^20^,^26^,^30^,^38^,^48^ (Strength 1; Evidence B)
- Diet control. Restriction of total calorie intake (25 kcal/kg per day is recommended) or reducing the calorie intake of the present diet by 500–1000 kcal/day^20^,^26^,^30^,^38^,^48^ (Strength 1; Evidence A)
- Weight loss. A loss of at least 3%–5% body weight appears necessary to improve steatosis, but greater weight loss (up to 10%) may be needed to improve necroinflammation^49^ (Strength 1; Evidence B)
- Weight loss rate. Weight loss of >1.6 kg/week, which may aggravate a fatty liver, should be avoided^50^ (Strength 2; Evidence B)

### Anti-obesity medications and bariatric surgery

If patients fail to reach a >5% weight reduction after changing their lifestyle for 6–12 months, the use of medications, such as orlistat, for secondary prevention should be considered (Strength 2,Evidence B).^20^,^26^,^30^,^38^,^48^,^51^ Orlistat has been reported to improve ALT and steatosis evaluated by ultrasonography.^52^ However, there another study reported that orlistat did not improve body weight or liver histology.^53^ The safety of anti-obesity drugs remains to be determined. According to the guidelines of the American Association for the Study of Liver Diseases (AASLD),^35^ upper gastrointestinal bariatric surgery may be considered in patients with morbid obesity who do not respond to weight-reducing medications and in whom there are no contraindications for the procedure. However, experience with bariatric surgery in the Chinese population is limited and it is premature to consider foregut bariatric surgery as a safe and effective option for the specific treatment of NASH in China (Strength 1; Evidence C).^20^,^26^,^30^,^38^,^48^

### Improve insulin resistance and metabolic status

It is recommended that metabolic risk factors and associated complications be actively treated in NAFLD patients with appropriate medications. It is recommended that NAFLD patients with no evidence of liver damage (e.g. serum transaminase greater than threefold the upper limit of normal), hepatic insufficiency, or decompensated cirrhosis use angiotensin receptor blockers, insulin sensitizers (e.g. metformin and pioglitazone), and statins to improve blood pressure, glucose and lipid metabolism, and atherosclerosis, respectively (Strength 1; Evidence B).

Because insulin resistance plays an important role in the pathogenesis of NAFLD, insulin sensitizers (e.g. metformin and thiazolidinediones) could be the most promising drugs for the treatment of NAFLD. However, a recent meta-analysis concluded that 6–12 months of metformin plus lifestyle intervention did not improve aminotransferases and liver histology compared with lifestyle intervention alone.^11^ Thus, metformin is not recommended as a specific treatment for NAFLD patients (Strength 1; Evidence A). Studies of the effect of pioglitazone show an improvement of aminotransferases, steatosis, ballooning, and inflammation to a certain degree in NASH patients.^54^–^57^ Pioglitazone treatment was also shown to significantly reduce (∼18%) the primary outcome of death, myocardial infarction, or stroke in patients.^58^ Accordingly, pioglitazone can be used for the treatment of steatohepatitis in NASH patients. However, there is also a higher rate of congestive heart failure with pioglitazone treatment compared with the control group (2.3% vs 1.8%, respectively; *P* = 0.002), thus caution must be exercised when considering its use in patients with impaired myocardial function.^58^ In addition, some of the adverse effects of thiazolidinediones, particularly weight gain and edema, need to be taken into consideration. Finally, the long-term safety and efficacy of pioglitazone in patients with NASH remain to be determined (Strength 1; Evidence B).

### Reduce additional insults to prevent aggravation of liver damage

Any medications that have potential hepatotoxicity, especially those that produce toxic metabolites through metabolism by the liver, should be avoided or used with caution. These medications include acetaminophen, amiodarone, valproic acid, and tamoxifen among others (Strength 1; Evidence B).^59^

### Use of liver protectants and antioxidants

Hepatocyte-protecting agents (e.g. ursodeoxycholic acid, polyene phosphatidylcholine, and n-3 polyunsaturated fatty acids) and antioxidants (e.g. vitamin E, vitamin C, and polyphenols) have been examined as potential treatments for NAFLD. There is yet insufficient evidence supporting the use of these medications as routine treatment for NAFLD/NASH patients.^20^,^26^,^30^,^38^,^48^,^60^ The use of one or two types of hepatic protectant (e.g. polyene phosphatidylcholine, vitamin E, silymarin, adenosylmethionine, and reduced glutathione^48^) is optional as adjunct therapy in the following patients: (i) NAFLD patients with NASH confirmed by liver histology; (ii) patients with significant liver injury, advanced hepatic fibrosis, or both, as evidenced by clinical features, laboratory findings, and imaging examination, including NAFLD patients with elevated serum transaminase, metabolic syndrome, or T2D; (iii) patients who are suspected of taking medications that may induce liver injury or those who have elevated serum transaminase levels during treatment; and (iv) patients with a coexisting hepatotropic viral infection or other liver disease. The duration of treatment is usually 6–12 months or longer (Strength 2; Evidence B). Of all the medications, vitamin E administered at a daily dose of 800 IU/day has shown definite effects in improving liver histology in non-diabetic NASH patients;^57^ thus, vitamin E can be considered as the first-line liver protectant (Strength 1; Evidence B).

## Follow-up

Once NAFLD is diagnosed, regular clinical assessment is required. It is recommended that NAFLD patients should be followed-up every 6 months after the implementation of lifestyle interventions and/or medications..^48^ The clinical assessments should include the following.

- Progression of NAFLD based on symptoms, liver enzymes, and liver ultrasonography. If ^1^H-MRS is available, it would be the preferred option to determine whether liver fat content has decreased following treatment.
- Metabolic parameters, specifically FBG, PBG or OGTT, HbA1c, and lipid profiles.
- For the patients with a poor response to treatment and bad progression of the disease, liver biopsy is recommended for the assessment of liver inflammatory grades and fibrosis scores.

Additional therapeutic strategies need to be discussed with liver disease experts or gastroenterologists (Strength 1; Evidence A).
